# Aminofluorination of 2-alkynylanilines: a Au-catalyzed entry to fluorinated indoles

**DOI:** 10.3762/bjoc.10.42

**Published:** 2014-02-20

**Authors:** Antonio Arcadi, Emanuela Pietropaolo, Antonello Alvino, Véronique Michelet

**Affiliations:** 1 Dipartimento di Scienze Fisiche e Chimiche, Università di L’Aquila, Via Vetoio- 67010 Coppito (AQ), Italy; 2 Dipartimento di Chimica, Sapienza, Università di Roma, P. le A. Moro 5, Roma, Italy; 3 Institut de Recherche de Chimie Paris, UMR 8247, Ecole Nationale Supérieure de Chimie de Paris, Chimie ParisTech, 11 rue P. et M. Curie, F-75231 Paris Cedex 05, France

**Keywords:** 2-alkynylanilines, fluorination, gold catalysis, indoles, tandem reactions

## Abstract

The scope and limitations of gold-catalyzed tandem cycloisomerization/fluorination reactions of unprotected 2-alkynylanilines to have access to 3,3-difluoro-2-aryl-3*H*-indoles and 3-fluoro-2-arylindoles are described. An unprecedented aminoauration/oxidation/fluorination cascade reaction of 2-alkynylanilines bearing a linear alkyl group on the terminal triple bond is reported.

## Introduction

**Scheme 1:**
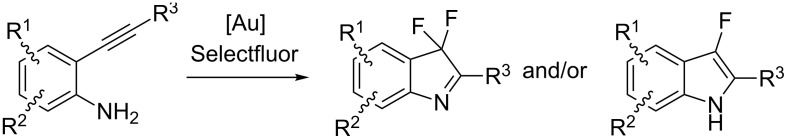
Cycloisomerization/fluorination of functionalized indoles.

Introducing fluorine atoms into organic molecules still faces challenges in organic synthesis [1]. Organofluorine compounds found growing use in various areas including pharmaceuticals, agrochemicals, and materials [2]. A significant proportion of pharmaceuticals are fluorinated derivatives because the inclusion of fluorine into organic compounds has been shown to improve properties such as solubility, bioavailability and metabolic stability, which are of great importance for the development of a large number of viable drug candidates [3]. Finding original methodologies for the selective preparation of fluorinated heterocyclic compounds is therefore still highly challenging [4–5]. The variation of the indole structure has been a field of high interest for a long time, considering the importance of this skeleton as a ubiquitous skeleton of pharmaceuticals and bioactive natural products [6–11]. The synthesis of 3-substiuted 3-fluorooxindoles has been described in the presence of Selectfluor as a commercial source of F^+^, starting from 3-substituted indoles, in acetonitrile/water. These derivatives have been used as key adducts for the indole biosynthesis mechanism as well as synthetic target for the development of novel medicinal agents [12]. Recently, 7-fluoroindole has been proposed as a potential candidate for the use in an antivirulence approach against persistent Pseudomonas aeruginosa infections [13]. Fluorine introduction in the benzene moiety of respective indoles was accomplished through a variety of methods [14]. 4-Fluoroindole derivatives have been prepared through nucleophilic attack on intermediate 4-indolediazonium salts [15]. The regioselective fluorination of the benzene ring of indole to give the important neurochemicals 4-fluoroserotonin and 4-fluoromelatonin was accomplished by means of a lithiation/fluorination sequence [16]. The validity of this latter strategy was also demonstrated for the fluorination at the 2-position of *N*-protected indoles by electrophilic fluorinating agents. Our literature search revealed that the access to C-3 fluorinated indole derivatives was less investigated. Fluorination of trialkylstannylindole derivatives with cesium fluoroxysulfate or Selectfluor was investigated for the synthesis of the corresponding 3-fluoroindoles [17]. A borane–tetrahydrofuran complex has been used to study the reduction of 3,3-difluoro-2-oxindoles to give the corresponding 3,3-difluoroindoines when electron-withdrawing groups were present as substituents in the benzene nucleus. The 3,3-difluoro-2-oxindoles were prepared by the reaction of appropriately substituted isatin derivatives with DAST [18]. Anodic fluorination of various *N*-acetyl-3-substituted indoles was successfully carried out to provide *trans*-2,3-difluoro-2,3-dihydroindoles which upon treatment with a base gave monofluoroindole derivatives or monofluoro-3*H*-indoles depending on the substituents at the 3-position [19]. More recently, the indole ring was difluorinated highly regioselectively at the C-3 carbon site with Selectfluor [20]. The C-3 monofluorinated indole derivatives were supposed to serve as intermediates in the transformation and can be isolated under suitable reaction conditions [21]. We envisaged that the aminofluorination of the ready available *o*-alkynylaniline derivatives should provide a viable alternative to the desired C-3 fluorinated indoles. Catalytic aminofluorination of alkenes and alkynes is receiving growing attention as efficient way to construct fluorinated heterocycles [22–23]. In particular, Au-catalyzed fluorination strategies by using Selectfluor as an electrophilic source of fluorine [24–31] can provide a powerful tool for building up nitrogen heterocycle derivatives. Fluorinated pyrrolidines [32] and fluorinated pyrazoles [33] have been synthesized from 1,ω-N-protected aminoalkynes and alkynyl phenylhydrazones, respectively. Propargyl amidines were converted into 5-fluoromethylimidazoles in the presence of Selectfluor under gold(I) catalysis [34–36] through a cascade cyclization/fluorination process [37]. Following our previous work on gold catalysts (Scheme 1) [38], we wish to report herein a comprehensive study on gold-catalyzed tandem cycloisomerization/fluorination reactions to access 3,3-difluoro-2-aryl-3*H*-indoles and 3-fluoro-2-arylindoles, putting the stress on the scope and limitations of such systems.

## Results and Discussion

### Optimization of the catalytic system

The substrate 2-{[4-(methoxy)phenyl]ethynyl}aniline (**1a**) was selected as a model substrate and was subjected to various conditions in the presence of Selectfluor as the electrophilic fluorine source. The results are compiled in Table 1. When the reaction of **1a** was carried out at room temperature with an excess of Selectfluor and water in CH_3_CN in the absence of any catalyst (Table 1, entry 1), no desired fluorinated indole was detected and degradation of the starting *N*-unprotected 2-alkynylaniline **1a** was observed, which excludes a fluorocyclization according to a direct electrophilic process [39]. When the same reaction was performed in the presence of [bis(trifluoromethanesulfonyl)imidate](triphenylphosphine)gold(I) catalyst [40] (5 mol %), the formation of the difluorinated 3*H*-indole **2a** was observed although in low overall yield (Table 1, entry 2). No traces of C–C bond formation, a competitive pathway in the presence of gold catalysts and Selectfluor [41–43] were observed. The yield of **2a** was increased to 35% when the reaction was carried out in the presence of 10 mol % of the gold catalyst (Table 1, entry 3). Various other parameters were modified to increase the reaction efficiency. The amount of water played a determinant role in CH_3_CN as the reaction medium (Table 1, entries 3–5). The presence of water is considered important both as a reagent and for helping the dissolution of Selectfluor in CH_3_CN. One of the most notable limitations on the use of Selectfluor is indeed its relative insolubility in commonly used organic solvents. Even in MeCN, the solvent of choice for many reactions with Selectfluor, its solubility is undesirably low and presents a limitation in its overall use as a fluorinating agent. The presence of a minimal amount of water has previously been reported to increase the yield of fluorination in the 5-position of mono- and nonbrominated 2-acylpyrroles with Selectfluor under microwave conditions [44]. The presence of larger amounts of water may nevertheless speed up the protodeauration of the indolylgold species derived from the gold-catalyzed aminoauration of **1a** to give the 2-substituted indole [39]. The reaction of **1a** was also evaluated in various solvents and proceeded nicely in EtOH compared to MeCN, 1,4-dioxane, and acetone (Table 1, entries 9, 11, and 12).

**Table 1:** Cycloisomerization/fluorination of 2-{[4-(methoxy)phenyl]ethynyl}aniline (**1a**).

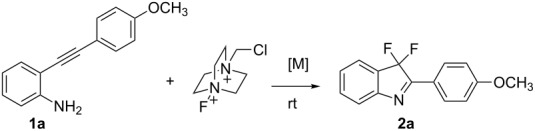					

Entry	[M] 10 mol %	Selectfluor (equiv)	solvent H_2_O (x equiv)	*t* [h]	Yield [%]^a^

1	–	2.5	CH_3_CN (10)	3	–
2	Ph_3_PAuNTf_2_^b^	2.5	CH_3_CN (10)	3	14
3	Ph_3_PAuNTf_2_	2.5	CH_3_CN (10)	3	35
4	Ph_3_PAuNTf_2_	2.5	CH_3_CN (50)	3	40
5	Ph_3_PAuNTf_2_	2.5	CH_3_CN (100)	3	17
6	Ph_3_PAuNTf_2_	1	CH_3_CN (100)	21	37
7	Ph_3_PAuNTf_2_	2.5	EtOH (0)	24	49
8	Ph_3_PAuNTf_2_	2	EtOH (100)	1.5	54
9	Ph_3_PAuNTf_2_	3	EtOH (100)	1.5	75
10	Ph_3_PAuNTf_2_	2	EtOH (50)	24	17
11	Ph_3_PAuNTf_2_	2	1,4-dioxane (100)	2	0
12	Ph_3_PAuNTf_2_	3	acetone (100)	2	0
13	AuCl	3	EtOH (100)	2	56
14	AuCl_3_	3	EtOH (100)	2	38
15	NaAuCl_4_	3	EtOH (100)	0.25	45
16	KAuCl_4_	3	EtOH (100)	2	53
17	PdCl_2_	3	EtOH (100)	2	0
18	PtCl_2_	3	EtOH (100)	2	0
19	CuCl_2_·2H_2_O	3	EtOH (100)	2	0
20	RuCl_3_·2H_2_O	3	EtOH (100)	2	0
21	AgNTf_2_	3	EtOH (100)	2	0

^a^Isolated yield. ^b^5 mol %.

In the presence of EtOH, we were pleased to find that the desired adduct **2a** was isolated in 75% yield (Table 1, entry 9). The addition of NaHCO_3_ [20,33] as a base to the reaction mixture failed to give **2a**, whereas it was successful for the preparation of fluorinated pyrazole, via the gold(I)-catalyzed tandem aminofluorination of 1-phenyl-2-(4-phenylbut-3-yn-2-ylidene)hydrazine. Other gold catalysts such as gold(III) species presented interesting results for the reaction beside lower yield than gold(I) catalyst (Table 1, entries 14–16 vs entries 9 and 13). Other transition metal catalysts such as PdCl_2_, PtCl_2_, CuCl_2_·2H_2_O, RuCl_3_·2H_2_O, or AgNTf_2_ were also tested, but did not give the desired difluorinated 3*H*-indole (Table 1, entries 17–21). We selected the PPh_3_AuNTf_2_ catalyst as a highly efficient complex and the cheaper NaAuCl_4_·2H_2_O catalyst and decided to confront their reactivity to various substrates.

We selected some derivatives (**1a**–**e**, **1g**, **1i** and **4a**,**b**) from literature [38–39,45–51] and synthesized them together with new functionalized 2-alkynylanilines to evaluate the efficiency of the gold catalytic system.

### Scope and limitations of the catalytic system

The prepared 2-substituted anilines were then engaged in the cycloisomerization/fluorination process in the presence of the Au(I) cationic catalyst or the Au(III) catalyst (Table 2, conditions A and B). The anilines **1b**–**1f** were subjected to conditions A and B at room temperature or refluxing ethanol. Under conditions A, the NaAuCl_4_·2H_2_O catalyst operated smoothly and Selectfluor (3 equiv) was added when full conversion of the gold(III)-catalyzed cyclization of **1** was observed. The use of the cationic PPh_3_AuNTf_2_ complex allowed in situ addition of Selectfluor. Both catalytic systems were efficient and depending on the substrate higher yields were obtained either in the presence of the Au(III) or Au(I) catalyst. The difluoro derivatives **2b**–**f** were isolated in moderate to very good yields. In the case of substrates **2b** or **2d** bearing an *o-*substituent group in the aryl moiety, better yields of these desired cyclized and functionalized derivatives were observed in ethanol at reflux (Table 2, entries 1/2 and comparison of 5 vs 6). The reaction conditions were compatible with a halogen substituent such as in **2e** (Table 2, entries 7 and 8), which was obtained in up to 67% yield. Gratifyingly the presence of 4-substituted 1*H*-pyrazole allowed the clean formation of the corresponding difluoroadduct **2f** in good isolated yields (Table 2, entries 9 and 10). One limitation was found when 2-substituted pyridylalkyne **1g** was subjected to conditions A (Table 2, entry 11). No reaction occurred and the starting material was recovered. A complex reaction mixture was obtained by reacting **1g** in EtOH at reflux for 1 h with an excess of Selectfluor (3 equiv) in the presence of Ph_3_PAuNTf_2_ catalyst (Table 2, entry 12).

**Table 2:** Cycloisomerization/fluorination reaction of 2-substituted anilines.

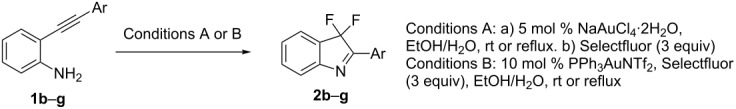						

Entry	Aniline	*t* [h]	Cond.	*T*	Product	Yield [%]^a^

1	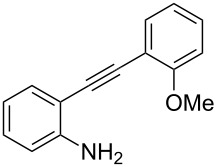 **1b**	1	A	reflux	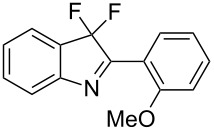 **2b**	60
2	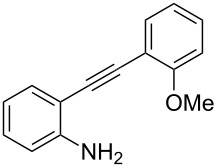 **1b**	0.75	B	reflux	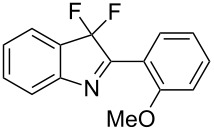 **2b**	74
3	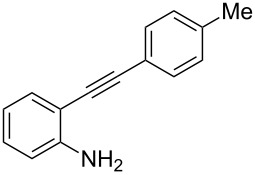 **1c**	24	A	rt	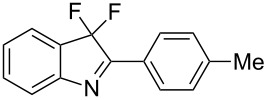 **2c**	83
4	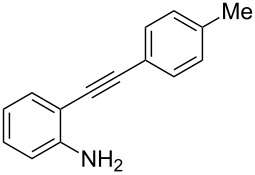 **1c**	1.5	B	rt	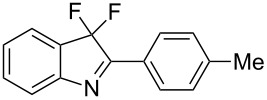 **2c**	57
5	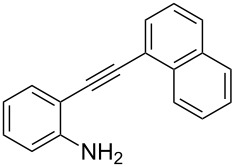 **1d**	43	A	rt	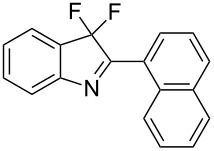 **2d**	76
6	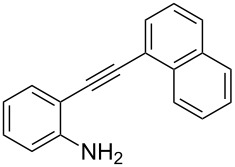 **1d**	0.75	B	reflux	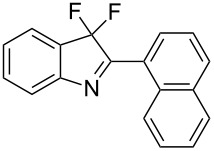 **2d**	85
7	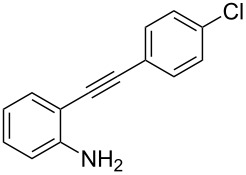 **1e**	24	A	rt	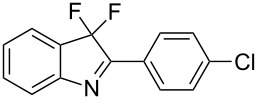 **2e**	67
8	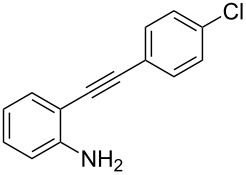 **1e**	2	B	reflux	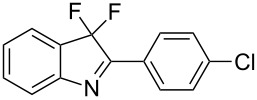 **2e**	40
9	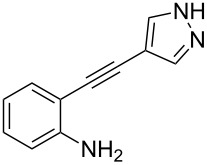 **1f**	3	A	reflux	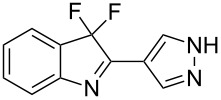 **2f**	61
10	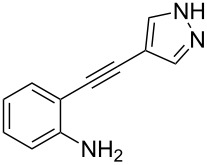 **1f**	2	B	reflux	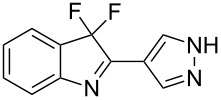 **2f**	65
11	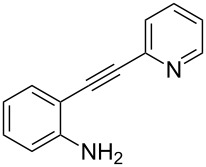 **1g**	24	A	reflux	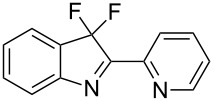 **2g**	–
12	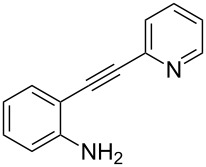 **1g**	1	B	reflux	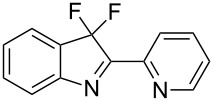 **2g**	–

^a^Isolated yield.

**Scheme 2:**
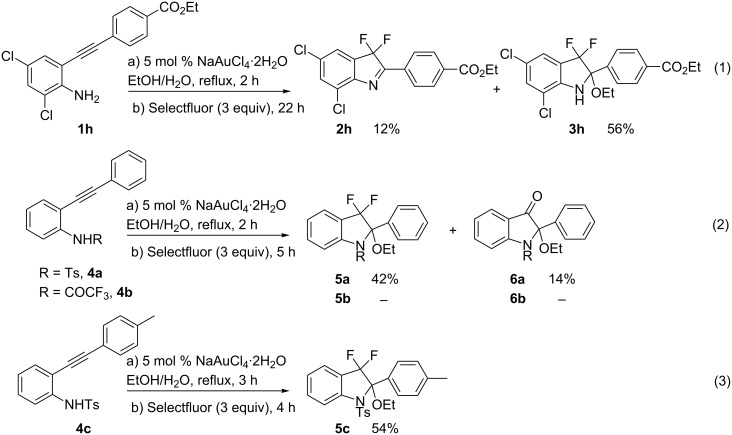
Synthesis of hemiaminal derivatives.

The case of aniline **1h** was particularly interesting as it showed that ethanol was not a fully inert solvent (Scheme 2, reaction 1). Indeed, when reacting aniline **1h** substituted with electron-withdrawing groups on both the aniline and the aryl moiety under Au(III) conditions, the desired product **2h** was accompanied by the hemiaminal difluoroadduct **3h**, which was isolated in 56% yield. The isolated **3h** spontaneously decomposed to give quantitatively **2h**. A similar trend was observed in the case of tosyl-protected aniline **4a** (Scheme 2, reaction 2). The reaction of the latter compound led to the formation of difluoro hemiaminal **5a** in 42% yield. Interestingly a novel derivative **6a** was also isolated in 14% yield. We also tested the reactivity of trifluoroacetyl-protected aniline **4b**, but no cyclization occurred and the starting material was recovered. In the case of the tolyl-substituted alkyne **4c**, the difluorinated product **5c** was the only isolated derivative in moderate 54% yield (Scheme 2, reaction 3).

**Scheme 3:**
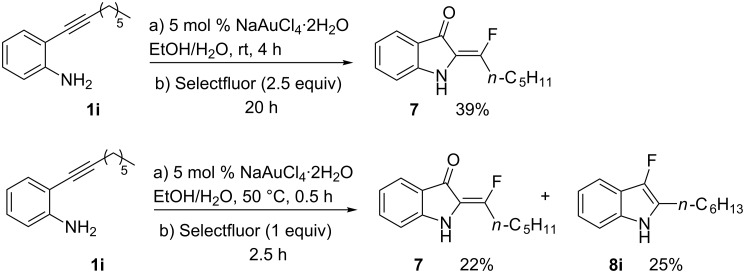
Reaction on *n*-hexyl-substituted derivative **1i**.

We also envisaged evaluating the influence of the alkynyl substituent of the aniline moiety. For this purpose, the Au(III)-catalyzed cycloisomerization/fluorination process was tested on *n*-alkyl-substituted derivative **1i** (Scheme 3). The reaction conducted at room temperature led to the (*E*)-2-(1-fluorohexylidene)indolin-3-one (**7**), whose structure and stereochemistry was confirmed by ^1^H NMR and NOESY experiments (see Supporting Information File 1), in 39% yield. Pleasingly, the monofluoro derivative **8i** was isolated in 25% yield by conducting the same reaction at 50 °C and in the presence of a lower amount of Selectfluor.

**Scheme 4:**
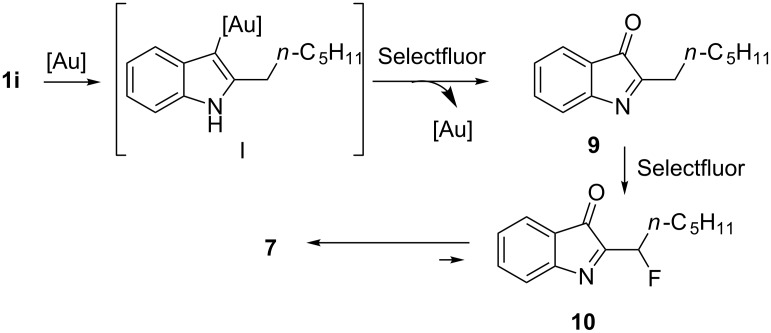
Mechanism rationale for the formation of **7**.

The mechanism for the formation of **7** (Scheme 4) may imply amino-auration of the alkyne **1i** to generate the indolyl–Au complex **I** according to the results observed in the tandem aminopalladation/oxidation process of azidoalkynes [52]. Then the C–Au bond is oxidized by Selectfluor [53–57] and would give the 2-hexyl-3*H*-indol-3-one (**9**). The formation of this latter derivative by the oxidation of 2-hexyl-1*H*-indole [58–62] or 3-fluoro-2-hexyl-1*H*-indole (**8i**) [63] cannot be ruled out. The following fluorination [64–67] of **9** led to 2-(1-fluorohexyl)-3*H*-indol-3-one (**10**), which tautomerizes to accomplish the stereoselective formation of **7**.

Considering the reactivity of anilines **1a**–**1i** in the presence of NaAuCl_4_·H_2_O complex in ethanol and the formation of **8** in the presence of 1 equivalent of Selectfluor, we decided to modify our initial procedure to selectively prepare 3-fluoro-2-aryl-indoles. We found that the cyclization of various unprotected anilines in acetonitrile followed by one-pot addition of Selectfluor in DMSO allowed the clean formation of monofluorinated derivatives and results are collected in Table 3.

**Table 3:** Cycloisomerization/fluorination reaction of 2-alkynyl-substituted anilines.

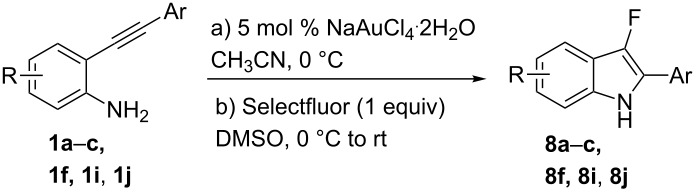				

Entry	Aniline	*t* [h] step a/b	Product	Yield [%]^a^

1	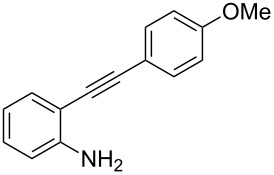 **1a**	1.5/1	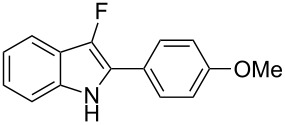 **8a**	41
2	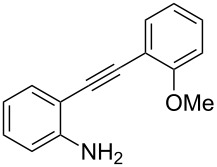 **1b**	0.2/3	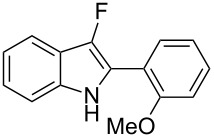 **8b**	37
3	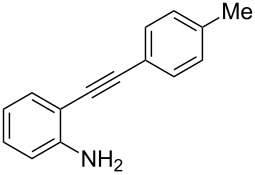 **1c**	1/1	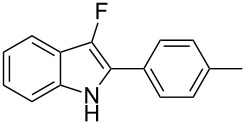 **8c**	55
4	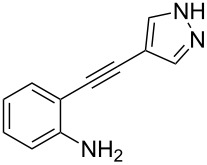 **1f**	0.3/1	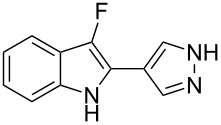 **8f**	20^b^
5	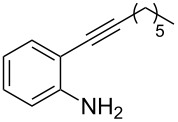 **1i**	3/4	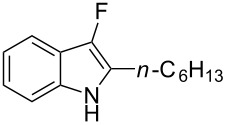 **8i**	40
6	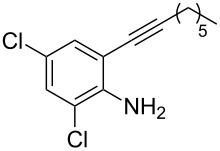 **1j**	1.5/6	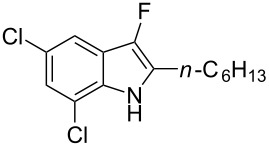 **8j**	85

^a^Isolated yield. ^b^40% of **2f** was also isolated.

With the optimal reaction conditions in hand, the substrate scope was examined. In the presence of the electron-rich aromatic groups on the terminal triple bond the desired products were isolated in moderate yields (Table 3, entries 1–3). In the presence of the 1*H*-pyrazolyl moiety the difluorination prevailed over the monofluorination process (Table 3, entry 4). With the 2-alkynylanilines bearing a linear alkyl group on the terminal triple bond, better results were observed in the presence of electron withdrawing groups in the aromatic ring of the aniline framework (Table 3, compare entry 5 with entry 6). It’s noteworthy that the preparation of 3-fluoroindoles is quite challenging. Because of overoxidation, the isolation of 3-fluoroindoles from 2-alkynylanilines has been reported to fail to occur [52] using previously developed silver catalysts [68]. In our cases, the fluorination reactions were conducted at 0 °C to avoid overoxidation processes.

## Conclusion

In conclusion, we have contributed to the development of one-pot gold-catalyzed aminofluorination of unprotected 2-alkynylanilines. The combination of a Au(I) or Au(III) complex associated to Selectfluor promotes the cycloisomerization/fluorination of non-protected aryl-substituted anilines at room temperature or refluxing ethanol. The reactions were found to be highly substrate- and solvent-dependent as different outcomes occur in ethanol or acetonitrile/DMSO mixture. The functionalized fluorinated indoles were isolated in moderate to very good yields. An unusual aminoauration/oxidation/fluorination cascade reaction was observed with 2-alkynylanilines bearing a linear alkyl group on the terminal triple bond. Further studies are in progress aimed to the selectivity control of the sequential gold-catalyzed oxidative cycloamination process of 2-alkynylanilines.

## Supporting Information

File 1 Experimental.
