# Supplementary material for: Aminofluorination of 2-alkynylanilines: a Au-catalyzed entry to fluorinated indoles
Source: Beilstein J Org Chem. 2014 Feb 20;10:449–58. doi: 10.3762/bjoc.10.42 (PMC3943415; doi:10.3762/bjoc.10.42)
Supplement: File 1 — Experimental. [file Beilstein_J_Org_Chem-10-449-s001.pdf]

# Supporting Information

for

# Aminofluorination of 2-alkynylanilines: a Au-catalyzed entry to fluorinated indoles

Antonio Arcadi*[1], Emanuela Pietropaolo[1], Antonello Alvino[2], Véronique Michelet*[3]

Address: [1]Dipartimento di Scienze Fisiche e Chimiche, Università di L'Aquila, Via Vetoio- 67010 Coppito (AQ), Italy, [2]Dipartimento di Chimica, Sapienza, Università di Roma, P. le A. Moro 5, Roma, Italy and [3]Institut de Recherche de Chimie Paris, UMR 8247, Ecole Nationale Supérieure de Chimie de Paris, Chimie ParisTech, 11 rue P. et M. Curie, F-75231 Paris Cedex 05, France

Email: Antonio Arcadi - arcadi@univaq.it; Véronique Michelet - veronique-michelet@chimie-paristech.fr

*Corresponding author

## Experimental

All manipulations were carried out under an argon atmosphere. $^1$H NMR and $^{13}$C NMR spectra were recorded on a Bruker AV 300 instrument. All signals were expressed as ppm ($\delta$) and internally referenced to residual protio solvent signals. Coupling constants (*J*) are reported in Hz and refer to apparent peak

multiplicities. $^{1}H$, $^{13}C$ NMR and mass spectroscopy data for compounds **2a**–**e** were described elsewhere [1].

**2-((1*H*-Pyrazol-4-yl)ethynyl)aniline (1f)**: To a solution of 4-iodopyrazole (0.365 g, 3.12 mmol) in THF (1 mL) and triethylamine (1.5 mL) were added $Pd(PPh_3)_2Cl_2$ (0.027 g, 0.039 mmol), copper(I) iodide (0.007 g, 0.039 mmol) and 2-ethynylaniline (0.500 g, 2.60 mmol). After 8 h of stirring at 50 °C under $N_2$, 100 mL of $H_2O$ and 100 mL of EtOAc were added. The organic layer was separated, and the aqueous layer was extracted with EtOAc. The combined organic layers were washed with water, dried over sodium sulfate, and concentrated. Purification of the residue by flash chromatography (eluent: hexane/EtOAc 70:30) afforded **1f** (0.211 g, 37% yield): Molecular formula: $C_{11}H_9N_3$; Mol. Wt.: 183.21 g/mol; purified by flash chromatography (Hexane/EtOAc 70:30); (0.211 g, 37 %); IR: (neat) $\nu_{max}$ 3182 (b), 2213, 1613, 1488, 749; $^{1}H$ NMR: (400 MHz, $CDCl_3$) δ 7.79 (s, 2 H), 7.38-7.35 (m, 1 H), 7.18-7.13 (m, 1 H), 6.76-6.72 (m, 3 H); $^{13}C$ NMR: (100 MHz, $CDCl_3$) δ 147.6, 136.4, 132.0, 129.6, 118.0, 114.4, 108.2, 103.2, 86.7, 85.4; MS (EI): *m*/*z*(%) = 182.8 (100) $[M-1]^+$, 183.8 (34).

**Ethyl 4-((2-amino-3,5-dichlorophenyl)ethynyl)benzoate (1h):** To a solution of ethyl 4-iodobenzoate (0.266 g, 0.97 mmol) in THF (9.0 mL) was added $Pd(PPh_3)_2Cl_2$ (0.017 g, 0.024 mmol), copper(I) iodide (0.015 g, 0.012 mmol) and 2,4-dichloro-6-ethynylbenzenamine (0.150 g, 0.81 mmol). After 4 h of stirring at 60 °C under $N_2$, 100 mL of $H_2O$ and 100 mL of EtOAc were added. The organic layer was separated, and the aqueous layer was extracted with EtOAc. The combined organic layers were washed with water, dried over

1. Arcadi, A.; Pietropaolo, E.; Alvino, A.; Michelet, V. *Org. Lett.* **2013**, *15*, 2766-2769.

sodium sulfate, and concentrated. Purification of the residue by flash chromatography (hexane/EtOAc 95:5) afforded **1h** (0.227g, 84%): Molecular formula: $C_{17}H_{13}Cl_2NO_2$; Mol. Wt.: 334.20 g/mol; purified by flash chromatography IR: (neat) $\nu_{max}$ 3315, 2193, 1721, 1604, 1462, 1272, 765; $^1$H NMR (400 MHz, $CDCl_3$) δ 8.05-803 (m, 2 H), 7.58-7.56 (m, 2H), 7.28-7.26 (m, 2 H), 4.39 (q, *J* = 7.13 Hz, 2 H), 3.95 (bs, 2 H), 1.41 (t, *J* = 7.13 Hz, 3 H); $^{13}$C NMR (100 MHz, $CDCl_3$) δ 165.9, 143.3, 131.4,130.4, 130.1, 129.8, 129.6, 126.8, 121.7, 119.2, 109.2, 95.4, 86.6, 61.2, 14.3; MS (EI): *m*/*z*(%) = 334.5 (62) and 332.4 (100) $[M]^+$.

**2,4-Dichloro-6-(oct-1-ynyl)aniline (1j)**: To a solution of 2-bromo-4,6-dichloroaniline (1.000 g, 4.16 mmol) in DMF (1 mL) and triethylamine (4.0 mL) were added $Pd(PPh_3)_2Cl_2$ (0.058 g, 0.083 mmol), copper(I) iodide (0.008 g, 0.042 mmol) and 1-octyne (0.550 g, 4.99 mmol). After 7 h of stirring at 50 °C under $N_2$, 100 mL of $H_2O$ and 100 mL of EtOAc were added. The organic layer was separated, and the aqueous layer was extracted with EtOAc. The combined organic layers were washed with water, dried over sodium sulfate, and concentrated. Purification of the residue by flash chromatography (eluent: hexane/EtOAc 99:1) afforded **1j** (0.774 g, 70% yield): Molecular formula: $C_{14}H_{17}Cl_2N$; Mol. Wt.: 270.20 g/mol; IR: (neat) $\nu_{max}$ 3489, 3387, 2219, 1610, 1464, 731; $^1$H-NMR: (400 MHz, $CDCl_3$) δ 7.19 (d, *J* = 2.35 Hz, 1 H), 7.16 (d, *J* = 2.35 Hz, 1 H), 4.55 (bs, 2 H), 2.48 (t, *J* = 7.10 Hz, 2 H), 1.67-1.57 (m, 2 H), 1.51-1.44 (m, 2 H), 1.36-1.33 (m, 4 H), 0.93 (t, *J* = 7.00 Hz, 3 H); $^{13}$C-NMR: (100 MHz, $CDCl_3$) δ 143.0, 129.9, 128.4, 121.4, 118.8, 110.9, 97.9, 75.5, 31.3, 28.7, 28.6, 22.5, 19.6, 14.0; MS (EI): *m*/*z*(%) = 270.8 (27) and 268.9 (44) $[M]^+$, 198.2 (96), 165.2 (99).

**3,3-Difluoro-2-(1*H*-pyrazol-4-yl)-3*H*-indole (2f):** $PPh_3AuNTf_2$ (0.038 g, 0.049 mmol) and Selectfluor (0.520 g, 1.47 mmol) were added to a solution of 2-((1*H*-pyrazol-4-yl)ethynyl)aniline **1f** (0.090 g, 0.49 mmol) in EtOH 4.9 mL)/$H_2O$ (0.87 mL). The mixture was stirred at reflux for 2.0 h. Then, the mixture was evaporated. Column chromatographic purification of the crude on silica gel (eluent: hexane/EtOAc 70:30) afforded the 3,3-difluoro-2-(1*H*-pyrazol-4-yl)-3*H*-indole (**2f**) (0.070 g, 65 %). Molecular formula: $C_{11}H_7F_2N_3$; Mol. Wt.: 219.19 g/mol; IR: (neat) $v_{max}$ 3500, 1579, 1261, 754 $cm^{-1}$; $^1H$ NMR: (400 MHz, acetone-$d_6$) δ 12.89 (bs, 1 H), 8.35 (s, 2 H), 7.68-7.49 (m, 3 H), 7.37-7.34 (m, 1 H); $^{13}C$ NMR: (100 MHz, acetone-$d_6$) δ 164.4 (t, *J* = 26.1 Hz), 154.0 (t, *J* = 9.5), 133.7 (t. *J* = 1.5 Hz), 129.9 (d, *J* = 237.6), 126.8 (t, *J* = 1.7 Hz), 123.0, 121.1, 111.6; $^{19}F$ NMR: (376 MHz, acetone-$d_6$) δ -118.0; MS (EI): *m*/*z* (%) = 218.9 $[M]^+$ (100), 126.0 (88).

**Ethyl 4-(5,7-dichloro-3,3-difluoro-3*H*-indol-2-yl)benzoate (2h):** $NaAuCl_4·2H_2O$ (0.004 g, 0.009 mmol) was added to a solution of ethyl 4-((2-amino-3,5-dichlorophenyl)ethynyl)benzoate (**1h**, 0.060 g, 0.18 mmol) in EtOH 1,8 mL)/$H_2O$ (0.32 mL). The mixture was stirred at reflux under $N_2$ atmosphere for 2.0 h until the complete conversion of the starting **1h** into the corresponding ethyl 4-(5,7-dichloro-1*H*-indol-2-yl)benzoate as monitored by GC-MS. Then, Selectfluor ( 0.191 g, 0.54 mmol) was added and the mixture after stirring under reflux for 22 h was evaporated. Column chromatographic purification of the crude on silica gel (eluent: hexane/EtOAc 95:5) afforded the ethyl 4-(5,7-dichlor-3,3-difluoro-3*H*-indol-2-yl)benzoate (**2h**, 0.010 g, 12%) and the ethyl 4-(5,7-dichloro-2-ethoxy-3,3-difluoroindolin-2-yl)benzoate (**3h**, 0.080 g, 56%).

**2h:** Molecular formula: $C_{17}H_{11}Cl_2F_2NO_2$; Mol. Wt.: 370.18 g/mol; IR: (neat) $v_{max}$ 1713, 1256, 706 $cm^{-1}$; $^1$H NMR: (400 MHz, $CDCl_3$) δ 8.28 (d, *J* = 8.84 Hz, 2 H), 8.18 (d, *J* = 8.84 Hz, 2 H), 7.54 (s, 1 H), 7.48 (s, 1 H), 4.43 (q, *J* = 7.13 Hz, 2 H), 1.43 (t, *J* = 7.13 Hz, 3 H); $^{13}$C NMR: (100 MHz, $CDCl_3$) δ 168.8 (t, *J* = 24.4 Hz), 165.6, 147.6 (t, *J* = 9.0 Hz), 134.5, 134.2, 133.6, 131.8, 129.9, 128.7, 128.4, 122.4 ( t, *J* = 259.0), 122.3, 61.4, 14.2; $^{19}$F NMR: (376 MHz, $CDCl_3$) δ -115.6; HRMS *m/z* (ESI) calcd for $C_{17}H_{12}Cl_2F_2NO_2$ $(M H)^+$ 372.0184 and 370.0213, found 372.0190 and 370.0688.

**Ethyl 4-(5,7-dichloro-2-ethoxy-3,3-difluoroindolin-2-yl)benzoate (3h):** Molecular formula: $C_{19}H_{17}Cl_2F_2NO_3$; Mol. Wt.: 416.25 g/mol; IR: (neat) $v_{max}$ 3351, 2981, 1716, 1277, 865 $cm^{-1}$; $^1$H NMR: (400 MHz, $CDCl_3$) δ 8.08 (dd, $J_1$ = 8.26 Hz, $J_2$ = 1.87 Hz, 2 H), 7.75 (dd, $J_1$ = 8.26 Hz, $J_2$ = 1.87 Hz, 2 H), 7.38 (s, 1 H), 7.33 (s, 1 H), 5.29 (s, 1 H), 4.39 (q, *J* = 7.11 Hz, 2 H), 3.48-3.34 (m, 2 H), 1.39 (t, *J* = 7.11 Hz, 3 H), 1.21 (t, *J* = 6.98 Hz 3 H); $^{13}$C NMR: (100 MHz, $CDCl_3$) δ 166.1, 148.0, 144.4 (t, *J* = 5.9 Hz), 139.3, 132.0, 131.6, 130.0, 129.6, 127.6, 125.8 (t, *J* = 296.8), 122.9, 116.1, 96.6 (dd, $J_1$ = 20.1, $J_2$ = 13.8 Hz), 61.19, 59.2, 15.3, 14.20.

**4-Methyl-*N*-(2-(*p*-tolylethynyl)phenyl)benzenesulfonamide (4c):** 2-(*p*-Tolylethynyl)aniline (**1c**, 0.144 g, 0.69 mmol), THF (1.8 mL), pyridine (0.545 g, 6.90 mmol) and tosyl chloride (0.263 g, 1.38 mmol) were stirred for 15 h. The crude was purified by column chromatography (hexane/EtOAc 95:5) to give the title compound **4c** (0.150 g, 60%); Molecular formula: $C_{22}H_{19}NO_2S$; Mol. Wt.: 361.46 g/mol; purified by flash chromatography (hexane/EtOAc 95:5); (0.150 g, 60%); IR: (neat) $v_{max}$ 3242, 2213, 1403, 1166, 1091, 754 $cm^{-1}$; $^1$H NMR: (400 MHz, $CDCl_3$) δ 7.67-7.60 (m, 3 H), 7.37-7.15 (m, 9 H), 7.07-7.03

(m, 1 H), 2.40 (s, 3 H), 2,33 (s, 3 H); $^{13}$C NMR: (100 MHz, $CDCl_3$) δ 143.9, 139.4, 137.4, 136.1, 131.9, 129.6, 129.4, 129.3, 127.2, 124.5, 120.2, 118.9, 114.8, 96.4, 83.0, 21.6, 21.5; HRMS *m/z* (ESI) calcd for $C_{22}H_{20}NO_2S$ $(M-H)^+$ 362.1215 found 362.1215.

**2-Ethoxy-3,3-difluoro-2phenyl-1-tosyl-3*H*-indole 5a:** $NaAuCl_4{\cdot}2H_2O$ (0.004 g, 0.009 mmol) was added to a solution of 4-methyl-*N*-(2-phenylethynyl) phenylbenzenesulfonamide **4a** (0.065 g, 0.19 mmol) in EtOH 1.9 mL)/$H_2O$ (0.33 mL). The mixture was stirred at reflux under $N_2$ atmosphere for 2 h until the complete conversion of the starting **4a**. Then, Selectfluor (0.202 g, 0.57 mmol) was added and the mixture after stirring under reflux for 5 h was evaporated. Column chromatographic purification of the crude on silica gel (eluent: hexane/EtOAc 95:5) afforded the 2-ethoxy-3,3-difluoro-2phenyl-1-tosyl-3*H*-indole (**5a**, 0.034 g, 42%) and the 2-ethoxy-2-phenyl-1-tosylindolin-3-one (**6a**, 0.011 g, 14%). **5a:** Molecular formula: $C_{23}H_{21}F_2NO_3S$; Mol. Wt.: 429.12 g/mol; IR: (neat) $v_{max}$ 2982, 1609, 1466, 704 $cm^{-1}$; $^1$H NMR: (400 MHz, $CDCl_3$) δ 7.84-7.82 (m, 1H), 7.57-7.52 (m, 2 H), 7.38-7.11 (m, 10 H), 3.80-3.59 (m, 2 H), 2.36 (s, 3 H), 1.32 (t, *J* = 6.50 Hz, 3 H); $^{13}$C NMR: (100 MHz, $CDCl_3$) δ 144.2, 143.3 (t, *J* = 6.6 Hz), 136.8, 133.8, 133.6, 129.2, 128.7, 128.0, 127.7, 127.5, 127.4, 127.2, 126.5 (t, *J* = 34.2 Hz), 124.2, 123.6, 121.0 (t, *J* = 254.1 Hz), 113.5, 99.5 (dd, $J_1$ = 21.8 Hz, $J_2$ = 8.9 Hz), 61.5, 21.5, 14.8; $^{19}$F NMR: (376 MHz, $CDCl_3$) δ -83.0 (d, J = 253.3), -107.1 (d, *J* = 253.3); HRMS *m/z* (ESI) calcd for $C_{23}H_{21}F_2NO_3SNa$ $(M-Na)^+$ 452.1086, found 452.1108.

**2-Ethoxy-3,3-difluoro-2-*p*-tolyl-1-tosyl-3*H*-indole (5c):** $NaAuCl_4{\cdot}2H_2O$ (0.008 g, 0.019 mmol) was added to a solution of 4-methyl-*N*-(2-(*p*-

tolylethynyl)phenyl)benzenesulfonamide (**4c**, 0.140 g, 0.39 mmol) in EtOH 3.9 mL)/$H_2O$ (0.66 mL). The mixture was stirred at reflux under $N_2$ atmosphere for 3 h until the complete conversion of the starting **4c**. Then, Selectfluor (0.414 g, 1.17 mmol) was added and the mixture after stirring under reflux for 4 h was evaporated. Column chromatographic purification of the crude on silica gel (eluent: hexane/EtOAc = 95:5) afforded the 2-ethoxy-3,3-difluoro-2-*p*-tolyl-1-tosyl-3*H*-indole (**5c**, 0.093 g, 54%). **5c:** Molecular formula: $C_{24}H_{23}F_2NO_3S$; Mol. Wt.: 443.51 g/mol; purified by flash chromatography (hexane/EtOAc 95:5); (0.044 g, 54 %); IR: (neat) $v_{max}$ 2981, 2926, 1609, 1465, 748 $cm^{-1}$; $^1H$ NMR: (400 MHz, $CDCl_3$) δ 7.83-7.81 (m, 1H), 7.56-7.52 (m, 2 H), 7.43-7.41 (m, 2 H), 7.27-7.24 (m, 2 H), 7.18-7.11 (m, 3 H), 6.99-6.97 (m, 2 H), 3.76-3.57 (m, 2 H), 2.37 (s, 3 H), 2.34 (s, 3 H), 1.30 (t, *J* = 6.90 Hz, 3 H); $^{13}C$ NMR: (100 MHz, $CDCl_3$) δ 144.1, 143.3 (t, *J* = 6.4 Hz), 138.6, 137.0, 133.5, 131.0, 129.2, 127.9, 127.5, 126.0, 125.8 (t, *J* = 235.2), 124.0, 123.6, 122.0 (t, *J* = 25.5 Hz), 118.4, 113.5, 99.7 (dd, $J_1$ = 21.8 Hz, $J_2$ = 8.6 Hz) 61.5, 21.5, 21.2, 14.7; $^{19}F$ NMR: (376 MHz, $CDCl_3$) δ -83.5 (d, *J* = 257.8), -107.5 (d, *J* = 254.4); HRMS *m/z* (ESI) calcd for $C_{24}H_{23}F_2NO_3SNa$ $(M\text{-}Na)^+$ 466.1259, found 466,1264.

**2-Ethoxy-2-phenyl-1-tosylindolin-3-one (6a):** Molecular formula: $C_{23}H_{23}NO_4S$; Mol. Wt.: 409.50 g/mol; IR: (neat) $v_{max}$ 2981, 1730, 1604, 1366, 658 $cm^{-1}$; $^1H$ NMR: (400 MHz, $CDCl_3$) δ 8.04 (d, *J* = 8.20, 1H), 7.75-7.61 (m, 2 H), 7.39-7.06 (m, 10 H), 3.57-3.43 (m, 2 H), 2.34 (s, 3 H), 1.29 (t, *J* = 6.96 Hz, 3 H); $^{13}C$ NMR: (100 MHz, $CDCl_3$) δ 196.5, 153.2, 144.2, 138.4, 136.7, 135.6, 129.3, 128.8, 128.2, 127.1, 126.1, 125.2, 123.4, 121.1, 114.3, 96.9, 60.6, 21.5, 14.8; HRMS *m/z* (ESI) calcd for $C_{23}H_{21}NNaO_4S$ $(M\text{-}Na)^+$ 430.1089, found 430,1012.

**Synthesis of (*E*)-2-(1-fluorohexylidene)indolin-3-one (7):** $NaAuCl_4 \cdot 2H_2O$ (0.009 g, 0.020 mmol) was added to a solution of 2-(oct-1-ynyl)aniline (**1i**, 0.095 g, 0.47 mmol) in EtOH 1.9 mL)/$H_2O$ (0.33 mL). The mixture was stirred at room temperature under $N_2$ atmosphere for 4 h until the complete conversion of the starting **1i**. Then, Selectfluor (0.418 g, 1.18 mmol) was added and the mixture after stirring for 20 h was evaporated. Column chromatographic purification of the crude on silica gel (eluent: hexane/EtOAc 90:10) afforded the (*E*)-2-(1-fluorohexylidene)indolin-3-one **7a** (0.044 g, 39%); Molecular formula: $C_{14}H_{16}FNO$; Mol. Wt.: 233.28 g/mol; IR: (neat) $v_{max}$ 3296, 1650, 1466, 743 cm$^{-1}$; $^1H$ NMR: (400 MHz, $CDCl_3$) δ 8.86 (s, 1H), 7.71-7.69 (m, 1 H), 7.36-7.34 (m, 2H), 7.17-7.13 (m, 1 H), 3.00-2-95 (m, 2 H), 1.81-1.77 (m, 2 H), 1.42-1.37 (m, 4 H), 0.92 (t, *J* = 7.14, 3 H); $^{13}C$ NMR: (100 MHz, $CDCl_3$) δ 191.9, 148.8, 146.2, 133.6, 127.3, 120.8, 119.4, 112.4, 40.4, 31.5, 24.2, 22.5, 13.9; $^{19}F$-NMR: (376 MHz, $CDCl_3$) δ -154.6; time-dependent NOESY (Nuclear Overhauser Effect Spectroscopy) and HOESY (Heteronuclear Overhauser Enhancement Spectroscopy) experiments were used to provide experimental measures of the conformation. NH exhibited a NOE with the aliphatic hydrogen of methylene 13 and with the aromatic hydrogen 6. Moreover a HOESY peak was observed between the fluorine and the methylene 13. The $^{19}F\{^1H\}$-HOESY NMR ($^1H$, 400.13 MHz) experiment was carried out with a mixing time of 400 ms and 32 scans were taken for each of the 256-t1 increments recorded. $^1H$–$^1H$ NOESY, NMR ($^1H$, 400.13 MHz): The duration of the mixing time was 600 ms and 128 scans were taken for each of the 256-t1 increments recorded. In all experiments, the recycle time was set to 2 s.

**Typical procedure for a two-step one-pot gold(III)-catalyzed synthesis of 3-fluoroindoles a–j: Synthesis of 3-fluoro-2-(4-methoxyphenyl)-1*H*-indole (8a):** $NaAuCl_4 \cdot 2H_2O$ (0.008 g, 0.020 mmol) was added to a solution of 2-[(4-methoxy)ethynyl]aniline (**1a**, 0.090 g, 0.40 mmol) in $CH_3CN$ (4 mL). The mixture was stirred at reflux for 1.5 h until complete disappearance of the starting material **1a**. The solution was cooled to 0 °C. DMSO (4 mL) and Selectfluor (0.142 g, 0.40 mmol) were added to the mixture. The reaction was allowed to stir at 0 °C for one hour, then gradually warmed up to room temperature. Then, 100 mL of $H_2O$ and 100 mL of EtOAc were added. The organic layer was separated, and the aqueous layer was extracted with EtOAc. The combined organic layers were washed with water, dried over sodium sulfate, and concentrated. Purification of the residue by flash chromatography (eluent: hexane/EtOAc 98:3) afforded 3-fluoro-2-(4-methoxyphenyl)-1*H*-indole (**8a**, 0.040 g, 41%). Molecular formula: $C_{15}H_{12}FNO$; Mol. Wt.: 241.09 g/mol; IR: (neat) $v_{max}$ 3432, 1599, 1252, 742 $cm^{-1}$; $^1H$ NMR: (400 MHz, acetone-$d_6$) δ 10.29 (s, 1 H), 7.82 (d, *J* = 8.9 Hz, 2 H), 7.57-7.53 (m, 2 H), 7.19-7.15 (m, 1 H), 7.03-7.01 (m, 3 H), 3.86 (s, 3 H) ; $^{13}C$ NMR: (100 MHz, acetone-$d_6$) δ 159.1, 131.1, 129. 9 (d, *J* = 238.8), 129.7 (d, *J* = 245.4), 126.8 (d, *J* = 5.2 Hz), 124.5, 122.5, 119.5 (d, *J* = 29.0 Hz), 115.7, 114.3, 112.9 (d, *J* = 44.4), 111,5, 54.7; $^{19}F$ NMR: (376 MHz,acetone-$d_6$) δ -174.9; MS (EI): *m*/*z* (%) = 241.2 $[M]^+$ (100).

**3-Fluoro-2-(2-methoxyphenyl)-1*H*-indole (8b):** Molecular formula: $C_{15}H_{12}FNO$; Mol. Wt.: 241.09 g/mol; purified by flash chromatography (hexane/EtOAc 95:5); (0.040 g, 37 %); IR: (neat) $v_{max}$ 3447, 1464, 1243, 744 $cm^{-1}$; $^1H$ NMR: (400 MHz, $CDCl_3$) δ 9.22 (s, 1 H). 8.09 (dd, $J_1$ = 6.10 Hz, $J_2$ =

1.70 Hz, 1 H), 7.66 (dd, $J_1$ = 6.10 Hz, $J_2$ = 1.70 Hz, 1 H), 7.37-7.05 (m, 6 H), 4.03 (s, 3 H); $^{13}$C NMR: (100 MHz, $CDCl_3$) δ 155.3, 142.3 (d, *J* = 247.0 Hz), 131.3 (d, *J* = 6.8 Hz), 128.9 (d, *J* = 12.1 Hz), 128.3, 127.3 (d *J* = 246. 8 Hz), 122.8, 121.7, 119.7, 118.5, 116.4 (d, *J* = 1.8 Hz), 111.7, 111.3 (d, *J* = 65.4 Hz), 111.1, 55.9; $^{19}$F NMR: (376 MHz, $CDCl_3$) δ -168.6; MS (EI): *m*/*z* (%) = 241.3 $[M]^+$ (100).

**3-Fluoro-2-*p*-tolyl-1*H*-indole 8c:** Molecular formula: $C_{15}H_{12}FN$; Mol. Wt.: 225.26 g/mol; purified by flash chromatography (hexane/EtOAc 95:5); (0.060 g, 55%); IR: (neat) $v_{max}$ 3431, 1599, 1456, 1347, 1238, 740 $cm^{-1}$; $^1$H NMR: (400 MHz, acetone-*$d_6$*) δ 10.36 (s, 1 H), 7.77 (d, *J* = 8.31 Hz, 2 H), 7.58-7-56 (m, 1 H), 7.40-732 (m, 3 H), 7.20-7.16 (m, 1 H), 7.12-7.08 (m, 1 H), 2.38 (s, 3 H); $^{13}$C NMR: (100 MHz, acetone-*$d_6$*) δ 142.7 (d, *J* = 244.2 Hz), 138.2, 131.0 (d, *J* = 239.0 Hz), 130.6, 126.4 (d, *J* = 5.3 Hz), 126.0, 125.7 (d, *J* = 57.3), 123.8, 120.8, 116.9 (d, *J* = 1.7 Hz), 112.6 (d, *J* = 5.2 Hz), 111.9 (d, *J* = 16.2 Hz), 21.3; $^{19}$F NMR: (376 MHz, acetone-*$d_6$*) δ -173.6; MS (EI): *m*/*z* (%) = 225.0 $[M]^+$ (100).

**3-Fluoro-2-(1*H*-pyrazol-4-yl)-1-*H*-indole (8f):** Molecular formula: $C_{11}H_8FN_3$; Mol. Wt.: 201.20 g/mol; purified by flash chromatography (Hexane/EtOAc 70:30); (0.020 g, 20 %); IR: (neat) $v_{max}$ 3418, 3158, 744 $cm^{-1}$; $^1$H NMR: (400 MHz, acetone-*$d_6$*) δ 10.71 (s, 1 H), 10.27 (s, 1 H), 8.09 (s, 2 H), 7.78-7.76 (m, 1 H), 7.67-7.65 (m, 1 H), 7.15-7.06 (m, 1 H); $^{13}$C NMR: (100 MHz, acetone-*$d_6$*) δ 140.2 (d, *J* = 240.0 Hz), 132.4 (d, *J* = 6.1 Hz), 131.1, 128.7, 121.9, 121.1 (d, *J* = 223.2 Hz), 119.6, 115.3 (d, *J* = 2.0 Hz), 114.6 (d, *J* = 22.4 Hz); $^{19}$F NMR: (376 MHz, acetone-*$d_6$*) δ - 176.1; MS (EI): *m*/*z* (%) = 201.1 $[M]^+$ (100).

**3-Fluoro-2-hexyl-1*H*-indole (8i):** Molecular formula: $C_{14}H_{18}FN$; Mol. Wt.: 219.30 g/mol; purified by flash chromatography (hexane/EtOAc 98:2); (0.044 g, 40 %); IR: (neat) $v_{max}$ 3387, 1459, 1260, 741 $cm^{-1}$; $^{1}H$ NMR: (400 MHz, acetone-$d_6$) δ 9.80 (s, 1 H), 7.46-7.01 (m, 4 H), 2.90-2.80 (m, 2 H), 2.07-2.05 (m, 2 H), 1.35-1.30 (m, 6 H), 0.89 (t, *J* = 7.10 Hz, 3 H); $^{13}C$ NMR: (100 MHz, acetone-$d_6$) δ; 136.5 (d, *J* = 243.8 Hz), 132.1, 122.1, 120. 6 ( d, *J* = 277.6 Hz), 119.9, 116.9 (d, *J* = 1.9 Hz), 111.9 (d, *J* = 5.2 Hz), 111.2 (d, *J* = 5.5 Hz), 32.2, 24.7, 24.6, 23.3, 23.2, 14.2; $^{19}F$ NMR: (376 MHz, acetone-$d_6$) δ - 181.5; MS (EI): *m*/*z* (%) = 219.0 $[M]^+$ (23), 148.1 (100).

**5,7-Dichloro-3-fluoro-2-hexyl-1*H*-indole (8j):** Molecular formula: $C_{14}H_{16}Cl_2FN$; Mol. Wt.: 288.18 g/mol; purified by flash chromatography (hexane/EtOAc 99:1); (0.097 g, 85 %); IR: (neat) $v_{max}$ 3387, 1459, 1260, 741 $cm^{-1}$; $^{1}H$ NMR: (400 MHz, acetone-$d_6$) δ 10.39 (s, 1 H), 7.30 (s, 1 H), 7.06 (s, 1 H), 2.79 (t, *J* = 7.90 Hz, 2 H), 1.57-1.52 (m, 2 H), 1.24-1.20 (m, 6 H), 0.85 (t, *J* = 6.76 Hz, 3 H); $^{13}C$ NMR: (100 MHz, acetone-$d_6$) δ 132.1 (d, *J* = 179.3 Hz), 132.0 (d, *J* = 191.8 Hz), 123.6, 119.6 (d, *J* = 233.6), 119.3, 116.4, 116.0, 110.6 (d, *J* = 4.5 Hz), 31.4, 29.5, 26.2, 22.3, 18.8, 13.4; $^{19}F$ NMR: (376 MHz, acetone-$d_6$) δ - 178.1; MS (EI): *m*/*z* (%) = 288.8 $[M]^+$ (7), 286.8 $[M]^+$ (13), 218.1 (76), 216.3 (100).

Time-dependent NOESY (Nuclear Overhauser Effect Spectroscopy) and HOESY (Heteronuclear Overhauser Enhancement Spectroscopy) experiments of compound **7**.

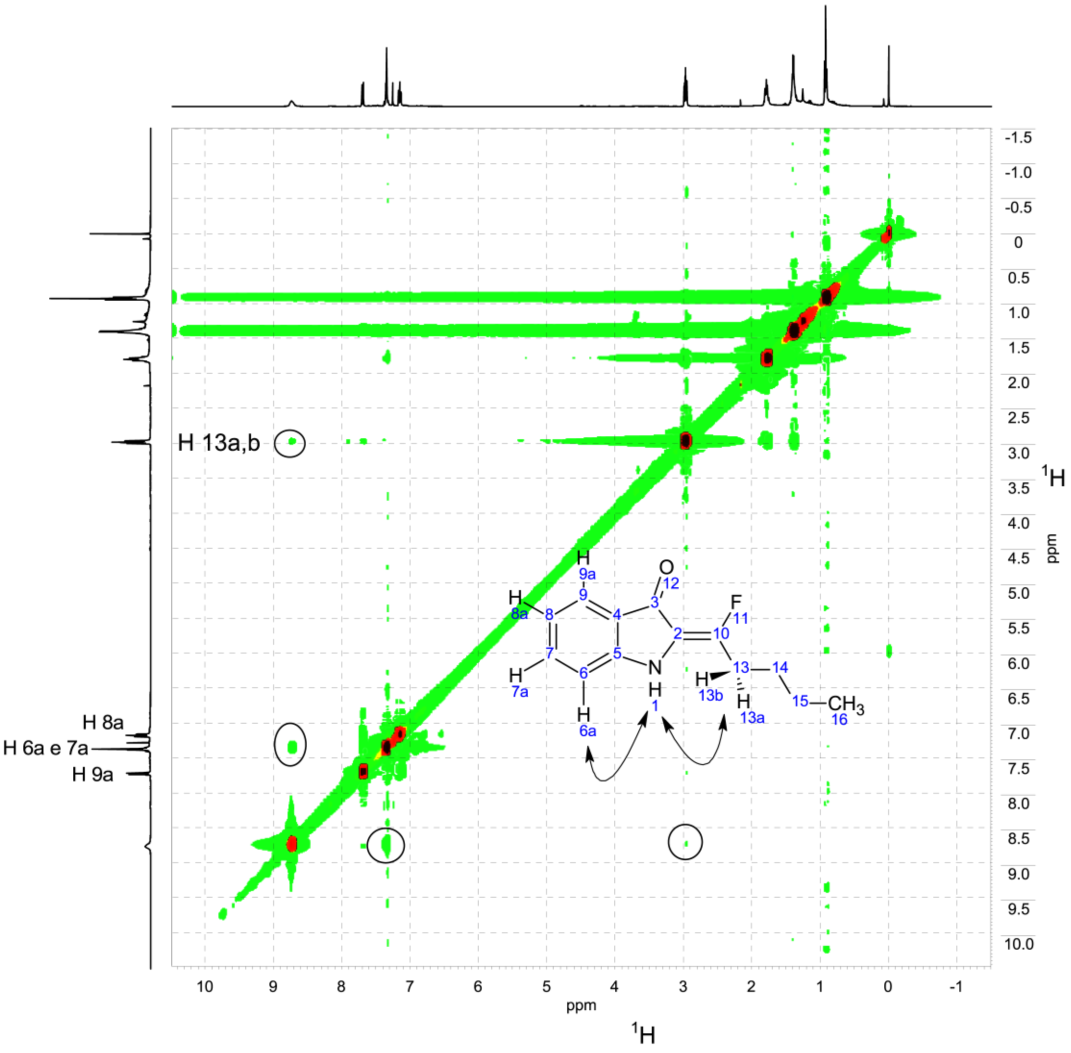

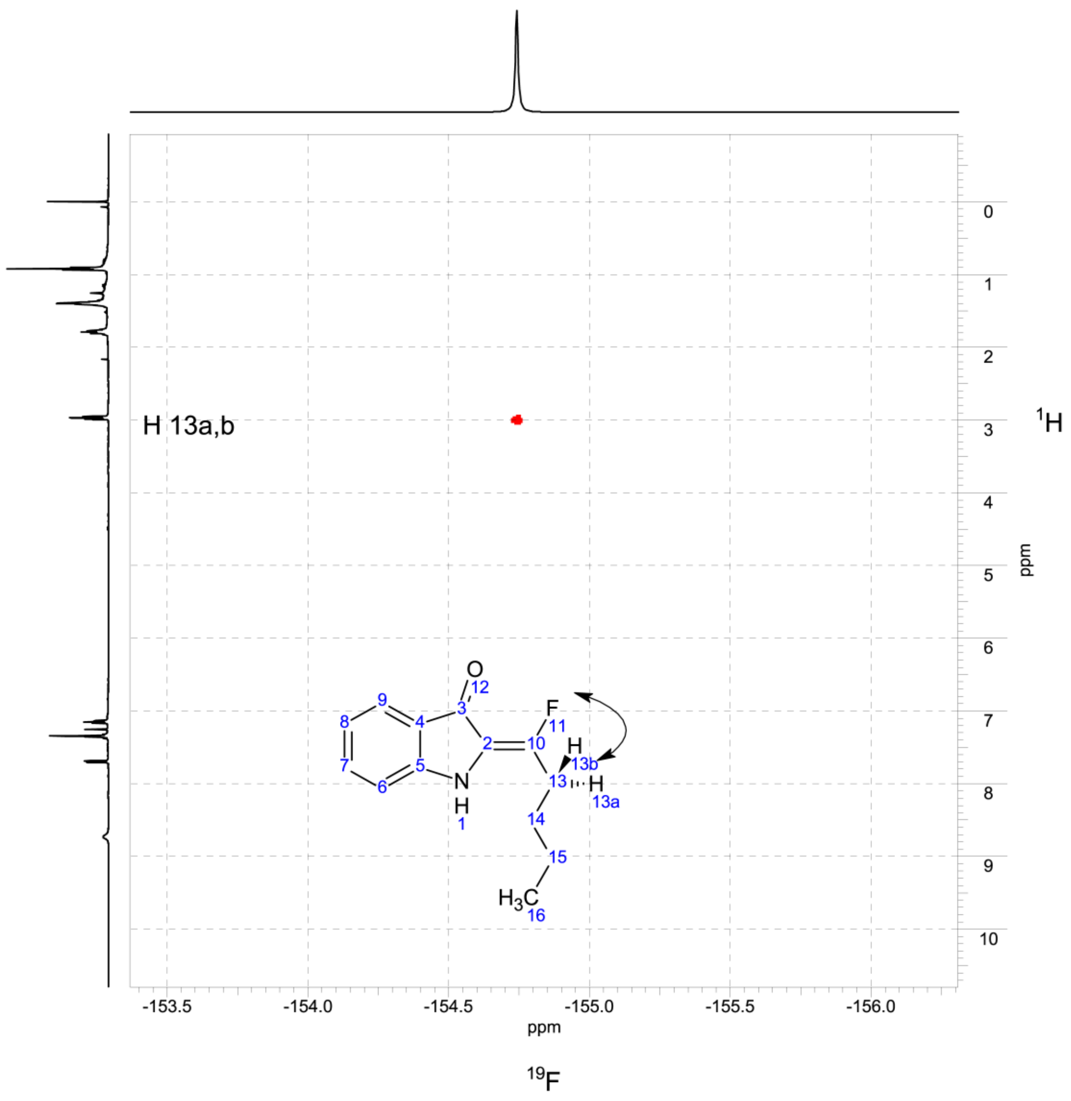
H 13a,b
0
1
2
3
4
5
6
7
8
9
10
ppm
1H
-153.5
-154.0
-154.5
-155.0
-155.5
-156.0
ppm
19F
O
12
3
9
8
4
2
10
F
11
H
13b
13
H
13a
7
5
6
N
H
1
14
15
H3C
16
